# Hyperinsulinemia is Associated with Increased Soluble Insulin Receptors Release from Hepatocytes

**DOI:** 10.3389/fendo.2014.00095

**Published:** 2014-06-19

**Authors:** Marcia Hiriart, Carmen Sanchez-Soto, Carlos Manlio Diaz-Garcia, Diana T. Castanares, Morena Avitia, Myrian Velasco, Jaime Mas-Oliva, Marina Macias-Silva, Clicerio González-Villalpando, Blanca Delgado-Coello, Marcela Sosa-Garrocho, Román Vidaltamayo, Deyanira Fuentes-Silva

**Affiliations:** ^1^Departamento de Neurodesarrollo y Fisiología, División de Neurociencias, Instituto de Fisiología Celular, Universidad Nacional Autónoma de México, Mexico City, Mexico; ^2^Centro Médico ABC, Mexico City, Mexico; ^3^Instituto de Ecología, Universidad Nacional Autónoma de México, Mexico City, Mexico; ^4^Departamento de Bioquímica y Biología Estructural, Instituto de Fisiología Celular, Universidad Nacional Autónoma de México, Mexico City, Mexico; ^5^Departamento de Biología Celular y del Desarrollo, Instituto de Fisiología Celular, Universidad Nacional Autónoma de México, Mexico City, Mexico; ^6^Unidad de Investigación en Diabetes, Instituto Nacional de Salud Pública, Cuernavaca, Mexico; ^7^Departamento de Ciencias Básicas, Universidad de Monterrey, Monterrey, Mexico; ^8^Departamento de Biomacromoléculas, Instituto de Química, Universidad Nacional Autónoma de México, Mexico City, Mexico

**Keywords:** diabetes mellitus type 2, hyperinsulinism, insulin resistance, metabolic syndrome, protease

## Abstract

It has been generally assumed that insulin circulates freely in blood. However it can also interact with plasma proteins. Insulin receptors are located in the membrane of target cells and consist of an alpha and beta subunits with a tyrosine kinase cytoplasmic domain. The ectodomain, called soluble insulin receptor (SIR) has been found elevated in patients with diabetes mellitus. We explored if insulin binds to SIRs in circulation under physiological conditions and hypothesize that this SIR may be released by hepatocytes in response to high insulin concentrations. The presence of SIR in rat and human plasmas and the culture medium of hepatocytes was explored using Western blot analysis. A purification protocol was performed to isolated SIR using affinity, gel filtration, and ion exchange chromatographies. A modified reverse hemolytic plaque assay was used to measure SIR release from cultured hepatocytes. Incubation with 1 nmol l^−1^ insulin induces the release of the insulin receptor ectodomains from normal rat hepatocytes. This effect can be partially prevented by blocking protease activity. Furthermore, plasma levels of SIR were higher in a model of metabolic syndrome, where rats are hyperinsulinemic. We also found increased SIR levels in hyperinsulinemic humans. SIR may be an important regulator of the amount of free insulin in circulation. In hyperinsulinemia, the amount of this soluble receptor increases and this could lead to higher amounts of insulin bound to this receptor, rather than free insulin, which is the biologically active form of the hormone. This observation could enlighten the mechanisms of insulin resistance.

## Introduction

Insulin is the most important hypoglycemic hormone in mammals. It is secreted by pancreatic beta cells in response to glucose stimulation and enters the portal circulation before inflowing the general circulation. More than 50% of insulin binds to insulin receptors (IR) in the liver and is removed from the circulating blood. The remaining hormone reaches peripheral targets, where it predominantly promotes anabolic effects in muscle and adipose tissue.

The IR has been extensively studied for more than 40 years. It is an acidic protein with an isoelectric point of 4.0 that constitutes a heavily glycosylated disulfide-linked homodimer (1, 2). Each monomer is formed by an alpha subunit (130 kDa) and a beta subunit (95 kDa) (3). The entire alpha chain and a short sequence of the beta chain comprise the ectodomain; beta-subunits have membrane spanning regions and cytoplasmic tyrosine kinase (TK) domains (4). The IR belongs to a family of receptors with TK activity, and it is closely related to the type-1 insulin-like growth factor receptor (IGF-1R) (5).

Insulin receptors are synthesized as single polypeptides with a signal sequence that directs the full monomers to insert into the plasma membrane. During its translocation, internal disulfide bridges are formed. After a proteolytic cleavage by furin, the protein is separated into alpha and beta chains, which stays connected by one disulfide bridge. The monomers further dimerizes by the formation of two other disulfide bridges between the alpha chains (6–8). In addition, alpha subunits are heavily glycosylated (9, 10). The dimeric IR contains both high- and low-affinity binding sites for insulin.

Insulin receptors are ubiquitous proteins and are distributed in all tissues at different densities. However, the major targets of insulin are the liver, which expresses the highest levels of the receptor, as well as adipose and muscular tissues. The complex formed between IR and insulin is internalized and transported to endosomes, where insulin is degraded by the action of the insulin-degrading enzyme (IDE). This enzyme is present in all types of cells, and not only in those with a clear response to insulin. Down-regulation of the IR was first described in lymphocytes over 40 years ago (11). Recently, the ectodomains of receptors for different hormones, cytokines, and growth factors have been found to circulate in blood, including a soluble IR ectodomain that was found to be elevated in the plasma of patients with diabetes (12).

The presence of soluble insulin receptors (SIR) in the blood raises many questions about its pathophysiology. Firstly, which tissues are the main contributors to the circulating IR levels? Do soluble IRs are associated to pathological conditions like metabolic syndrome (MS)? And last but not least, which are the stimuli that promote IR release from these tissues?

Several cell lines, including the human hepatoma HepG2 cells, have been described to release IRs when facing high concentrations of insulin (13), a context that resemble the high levels of circulating insulin in insulin resistance states (14). Interestingly, these cells also release more SIR when stimulated with high glucose concentrations, indicating that this process can also be exacerbated in conditions that mimic diabetes mellitus (15). Furthermore, it has been proposed that insulin promotes the activity of a proteinase that mediates the release of IRs from hepatocytes (16). Interestingly, in spontaneous hypertensive rats (SHR), an increased proteinase activity causes insulin-receptor cleavage in insulin-sensitive tissues, accounting for a worsening in glucose homeostasis (17). Indeed, circulating IRs are positively associated with hyperglycemia, glycoalbumin, and glycated hemoglobin levels in diabetic patients (12), however, the causal relationships between SIR and insulin resistance are not well-understood.

On one hand, the reduction in the density of IRs in the plasma membrane of target tissues may affect glucose handling (17), indicating that insulin resistance is a byproduct of an impaired insulin signaling. Nevertheless, a more active role may be ascribed for the remaining circulating receptors, since the injection of exogenous IRs may cause glucose intolerance in mice (18). Noteworthy, IRs released from cultured cells preserve the ability to bind insulin with high affinity (13), a property that is also present, although with low affinity, in alpha–beta monomers, alpha chains, and the soluble dimeric ectodomain (5). The latter evidence suggests that SIR may bind insulin in the plasma and buffer the amount of insulin available to exert biological effects.

In this study, we explored the possibility that exposure to high insulin levels induces the release of the IR ectodomain (soluble IR; SIR) from hepatocytes. We demonstrate that exposure to a high, physiological concentrations of insulin (1 nmol l^−1^) induces the release of SIR into the incubation medium of hepatocytes from healthy, wild-type male rats. This effect could be partially prevented by inhibiting protease activity. To test if a similar process could be present in the whole animal, we used a rat model of MS (19, 20) that presents hyperinsulinemia. We observed that levels of circulating SIR were increased.

We also explored the presence of SIR in rat plasma and found that a fraction of the circulating insulin is bound to plasmatic proteins, mostly albumin, and SIR. Furthermore, SIR levels were also increased in human subjects during hyperinsulinemia episodes, compared to healthy human adults, in which SIR could also be detected, albeit at much lower levels.

By extending this scenario, it is possible that SIR increases and buffers part of the circulating insulin, preventing the development of hypoglycemia in healthy subjects, in response to high insulin levels. Moreover, during the hyperinsulinemic episodes in patients with MS and insulin resistance, despite to the high levels of insulin detected, a significant proportion of the hormone could not be biologically available because it is bound to SIR.

## Materials and Methods

### Reagents

Dulbecco’s modified Eagle’s medium (DMEM) culture media, glutamine, gentamicin, and antibiotic–antimycotic solution were purchased from Life Technologies Corp. (CA, USA). Collagen type IV, HEPES, dl-dithiothreitol (DTT), pig insulin, sodium bicarbonate, glucose, sucrose, l-proline, d-(+)-galactose, NaCl, trizma base, trizma hydrochloride, and glycine were obtained from Sigma-Aldrich (MO, USA). Acrylamide, *N*,*N′*-methylene-bis-acrylamide, *N*,*N*,*N*′,*N*′-tetramethylethylenediamine (TEMED), ammonium persulfate, Tween 20, protein standard of molecular weights, and the protein determination kit, based on the Bradford assay (Cat. 5000-002), were purchased from Bio-Rad (CA, USA). Amicon YM-3 filters and Immobilion-FL membrane were obtained from Millipore (MA, USA), fetal bovine serum (FBS) from Equitech (England), and ECL plus Western blotting detection systems from General Electric Healthcare (Buckinghamshire, UK).

### Animals

All methods used in this study were approved by the Animal Care Committee of the Instituto de Fisiología Celular, Universidad Nacional Autónoma de México. Animal care was to the “International Guiding Principles for Biomedical Research Involving Animals,” Council for International Organizations of Medical Sciences, 2010. Wistar rats were obtained from the local animal facility, maintained in a facility with a 12:12 h light–dark cycle (06:00-18:00), and allowed free access to standard laboratory rat diet and tap water.

### Rat model of metabolic syndrome

We used a model of MS in adult male Wistar rats as previously reported by our group (19). Briefly, MS was induced in 2-months-old rats by a hypercaloric diet over 8 weeks, which consisted of a 20% (w/v) sucrose solution as drinking water. The control group received plain water.

### Study subjects

Human blood samples were donated by Dr. Clicerio González from the Unidad de Investigación en Diabetes, Instituto Nacional de Salud Pública. Patients were diagnosed with diabetes mellitus and had the disease for 1 year or more. As controls, we used the blood of young healthy Mexican volunteers without a history of familial diabetes and with a body mass index (BMI) ranging from 19 to 23. All participants in the Mexico City Diabetes Study gave an informed consent previously approved by the Institutional Review Board (IRB) of the Diabetes Center of the Hospital ABC from Mexico City, which provided ethical and regulatory guidance for research in the institution.

The IRB guaranteed the protection of the rights, welfare, and well-being of human research participants recruited for projects conducted by investigators of the institutions. To assure the highest ethical standards for human research protection, every protocol adhered to the ethical principles outlined in the Belmont Report and they were also implemented in accordance to the terms based on the code of the Mexican federal regulations 45 CFR 46 and 21 CFR 50 and 56. The IRB is registered at the Comisión Federal para la Protección de Riesgos Sanitarios, COFEPRIS (Federal Commision for the Protection of Sanitary Risks) and operated in accordance to the norms and regulations of the Secretaría de Salud (Health Department) of Mexico, as well as the National Institutes of Health, USA.

### Plasma samples

Peripheral blood from study subjects was collected in heparinized tubes and centrifuged at 302 × *g* at 5°C for 15 min. The supernatant was recovered and stored at −70°C for further use.

### Glucose and insulin determination

Glucose and insulin levels were measured using the commercials kits QuantiChromTM Glucose Assay Kit (Bioassay Systems, Hayward, CA, USA, Cat. DIGL-100) and Ultrasensitive Rat Insulin ELISA (Mercodia, Uppsala, Sweden; Cat. 10-1173), respectively, following the manufacturer’s instructions.

### Primary cultures

Rat hepatocytes were isolated using the method of liver digestion using portal vein collagenase perfusion as previously described (21). Hepatocytes were resuspended in sterile phosphate-buffered saline (PBS), diluted (v/v) with an isosmotic Percoll solution (45 mL Percoll/4.5 ml of 10× Hanks balanced salt solution and 0.5 ml of 1 mol l^−1^ Hepes) and centrifuged at 800 rpm for 5 min. Cell viability ≥85% was estimated by trypan blue exclusion. Hepatocytes were resuspended in DMEM plus 0.02% (w/v) bovine serum albumin, 3 mmol l^−1^ Hepes, 1 mmol l^−1^ sodium pyruvate, 6 mmol l^−1^ sodium bicarbonate, 1 mg/ml galactose, 0.2 mmol l^−1^ proline, 4 mmol l^−1^ l-glutamine, 10% (v/v) FBS, 5 μg/ml insulin–transferrin–sodium selenite (ITS), streptomycin (100 μg/ml), penicillin (100 units/ml), gentamicin (100 units/ml), and fungizone (0.25 μg/ml). For primary culture, hepatocytes were plated (2.5 × 106) in culture dishes coated with type-1 collagen (1 mg/ml) and allowed to attach over 3 h in attachment medium with 10% FBS under a 5% CO_2_/95% air atmosphere at 37°C (22). Under these conditions of culture, the hepatocytes were maintained in a quiescent state. After 3 h, the medium was replaced with 10.6 mmol l^−1^ glucose, insulin-free DMEM culture medium (without FBS or albumin), or supplemented with four insulin concentrations (50, 100, 500, or 1000 pmol l^−1^). Conditioned media from hepatocytes were collected at 100 h post-incubation and then centrifuged (302 × *g* at 5°C for 15 min), concentrated in Amicon centrifugal filters with a cutoff of 3 kDa at 2150 × *g* and 4°C for 10 min, and stored at −70° C.

### Immunoprecipitation

Total protein (2 mg) from plasma were incubated overnight with 5 μl of a monoclonal anti-alpha-IR antibody (200 μg/ml, Antigenix) or anti-insulin antibodies (200 μg/ml, cat, sc-9168, Santa Cruz Biotechnology) and then with 60 μl of protein-G agarose (Upstate, NY, USA) for 2 h and washed with 0.1% (v/v) TNTE buffer (20 mmol l^−1^ Tris–HCl, pH 7.5, 120 mmol l^−1^ NaCl, 0.5% (v/v) Triton-X 100, and 1 mmol l^−1^ EDTA). Samples were then centrifuged (9677 × *g* at 4°C for 1 min) and the pellets were diluted in 50 μl sample buffer for electrophoresis.

### Native gel electrophoresis

Samples were mixed with Native Charge Buffer (Bio-Rad) at a 1:1 ratio (v/v) and 20–40 μg of total protein were loaded on a 5–20% (w/v) acrylamide–bisacrylamide gradient Tris–glycine gel.

### SDS-PAGE electrophoresis

Samples were mixed with Laemmli buffer (Bio-Rad) plus 50 μmol l^−1^ DTT at a 1:1 ratio (v/v) and then incubated in boiling water for 5 min. Samples (20–40 μg) were then added to each well of a 3% (w/v) stacking, 7.5% (w/v) resolving acrylamide–bisacrylamide Tris–glycine gel with 0.1% (w/v) SDS.

### Western blot

Proteins were transferred from acrylamide gels to polyvinylidene fluoride (PVDF) membranes and the membranes were further stained with Ponceau solution (0.2% v/v) to corroborate the transfer efficiency. PVDF membranes were incubated overnight with primary antibodies against alpha-IR, albumin, or insulin diluted in 20 mmol l^−1^ Tris–HCl, 8 g/l NaCl, and 5% (w/v) non-fat dry milk. The blots were then incubated using a second antibody conjugated to horseradish peroxidase, and finally the membranes were processed using the ECL plus chemiluminescent kit to visualize the protein bands. Densitometry was performed on each band signal using the image analyzer software ImageJ from NIH, USA.

For further information about the antibodies, see the Table 1.

**Table 1:** **Antibody information**.

Antibody	Laboratory		Dilution	Cat. No.
Insulin (H-6)	Santa Cruz Biotechnology	Polyclonal	1: 1000	Sc-9168
Alpha-IR (N-20)	Santa Cruz Biotechnology	Polyclonal	1: 500	Sc-710
Beta-IR (H-70)	Santa Cruz Biotechnology	Polyclonal	1: 1000	Sc-20739
Beta-IR (C-19)	Santa Cruz Biotechnology	Polyclonal	1: 1000	Sc-711
BSA	Millipore	Polyclonal	1:1000	07-248
Alpha-IR	Antigenix	Monoclonal	1:250	MC810888AF
Alpha-IR	USBiological	Monoclonal	1:250	17661-28A

### Purification procedures

Each run consisted of 1 ml of samples from hepatocyte culture supernatants that were previously concentrated in Amicon centrifugal filters with a cutoff of 30 kDa at 2150 × *g* and 4°C for 10 min. Then, 50 mg of protein (50% of column capacity) were resuspended in charge buffer (20 mmol l^−1^ Tris–HCl, pH 7.4, and 500 mM NaCl), filtered with 0.22 μm Millipore membrane filter (Millipore, Billerica, MA, USA), and injected onto a concanavalin-A affinity chromatography column. Elution was performed using 20 mmol l^−1^ Tris–HCl, pH 7.4, and either 600 mmol l^−1^ glucose or 300 mmol l^−1^ beta-methyl-d-mannopyranoside (Sigma).

The retained fractions were pooled and 1 ml aliquots were subjected to gel filtration for insulin-receptor purification on a HiLoad 16/20 Superdex 200 column equilibrated and eluted with 50 mmol l^−1^ Tris–HCl and 500 mmol l^−1^ NaCl at pH 8 in an Akta FPLC system (GE Healthcare, USA) at a flow rate of 0.8 ml/min. Fractions of interest were pooled and desalted by successive washing steps of three volumes of 50 mmol l^−1^ Tris–HCl buffer each followed by centrifugation in Amicon filters with a 3 kDa cutoff. Then, 1 ml aliquots were charged into a MonoQ HR 5/5 column pre-equilibrated with 50 mmol l^−1^ Tris–HCl buffer, and the sample components were eluted at a flow rate of 0.5 ml/min and a gradient of 2.5 grades/min of a NaCl solution (1 mol l^−1^). For each purification step, the presence of SIR in the collected fractions was monitored by Western blot.

### Insulin measurement

Overnight (12 h) fasted rats were anesthetized and central blood was collected using a sterile syringe. Blood was placed in heparinized tubes and centrifuged at 8870 × *g* (4°C for 19 min). The supernatants were recovered and stored at −20°C before use. Insulin levels in plasma were measured using an Ultrasensitive Rat Insulin ELISA kit (Mercodia, Uppsala, Sweden; Cat. 10-1173), following the manufacturer’s instructions.

### Protein-bound insulin determination in plasma

Rat plasmas were centrifuged at 2319 × *g* for 15 min in Amicon Ultra-4 filters with a cutoff of 30 kDa (Merck Millipore, Billerica, MA, USA). Insulin concentration in the fraction containing proteins above 30 kDa was determined using the above-mentioned ELISA kit and adjusting for the concentration factor in each sample.

### Reverse hemolytic plaque assay for SIR

The reverse hemolytic plaque assay (RHPA) is a useful method to study secretion in endocrine cells (23, 24). This method was modified to measure the release of IRs from hepatocyte membranes. Briefly, after 1 day of culture in attachment medium, the hepatocytes were detached from culture dishes and mixed with equal volumes of *Staphylococcus aureus* protein-A-coated sheep erythrocytes previously conjugated with alpha-IR (N20) antibodies. The mixture was introduced to collagen-coated Cunningham chambers to promote cell attachment, and then incubated overnight in insulin-free medium, medium containing 1 nmol l^−1^ insulin, or medium with 1 nmol l^−1^ insulin and alpha2-macroglobulin (1.5 mg/ml). We then applied guinea pig complement diluted (1:40) in Hanks balanced salt solution and supplemented with 200 units/ml penicillin G, 200 mg/ml streptomycin, and 0.1% (w/v) BSA to Cunningham chambers and incubated them for 1 h. SIR release was determined by the presence of hemolytic plaques around the hepatocytes, which resulted from the complement-mediated lysis of erythrocytes expressing SIR–alpha-IR (N20) antibody complexes bound to protein A.

To measure SIR release from hepatocytes, the hemolysis plaques were observed under an inverted light microscope (Nikon, model TMS-F) and their diameters were measured using a calibration bar. At least 30 cells were counted per duplicate in each experimental condition for each culture, and the averages of the immunoplaque areas were calculated.

### Data analysis

The statistical analysis was performed using the software GraphPad InStat version 3.00 (GraphPad Software, San Diego, CA, USA). An unpaired Student’s *t*-test was used for comparisons between groups, applying a Welch correction in case of variance heterogeneity. Correlations between insulin and soluble insulin-receptor levels were performed using a Pearson test. Graphics were constructed using Origin version 7 (OriginLab Corporation, Northampton, MA, USA). Data are represented as mean ± standard error of the mean (SEM).

## Results

### Insulin is bound to proteins in the circulation

**Figure 1:**
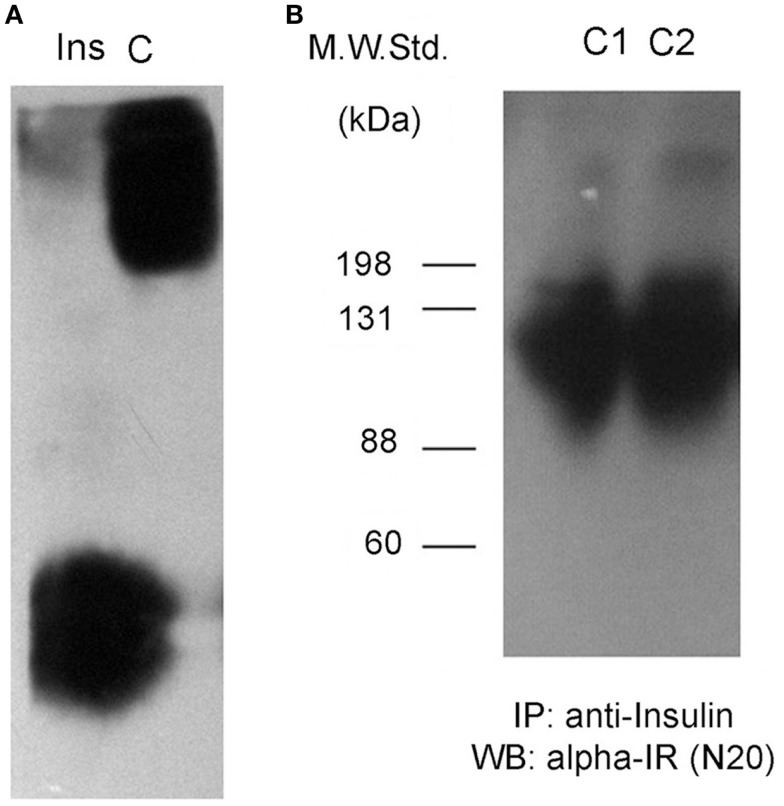
**Insulin interacts with a soluble receptor in plasma from control rats**. **(A)** Western blots against insulin after native gel electrophoresis from an insulin standard (Ins) and a plasma sample from an adult male Wistar rat (C). Insulin staining for the plasma sample is not located at the same position as the insulin standard, displaying less migration in the gel for plasma insulin. **(B)** Insulin-receptor (alpha-IR) staining by Western blot after denaturing gel electrophoresis of previously insulin-immunoprecipitated plasma samples from two control rats (C1 and C2).

We first explored the formation of protein complexes containing insulin in plasma samples from control male Wistar rats. We electrophoresed whole plasma samples on native gels and analyzed protein complexes by Western blot probed with an anti-insulin antibody (Figure 1A, see Materials and Methods). Strikingly, insulin-positive bands from normal plasma migrated at a higher molecular weight than purified, recombinant insulin standards. This result could be due to the formation of complexes between insulin and other circulating proteins. A soluble form of the IR has been described in diabetic patients (12), and thus we tested if IR was present in control animals.

**Figure 2:**
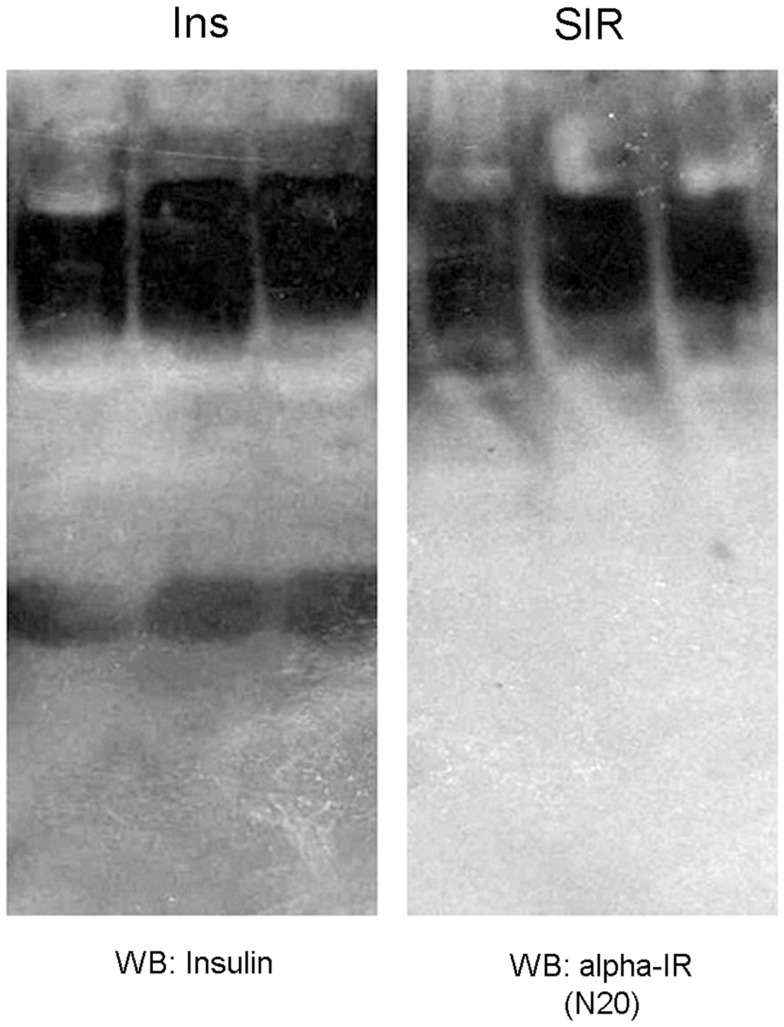
**Western blot against insulin (Ins) and a soluble insulin receptor (SIR) after native gel electrophoresis from plasma samples of three adult male Wistar rats**. As observed, insulin and SIR bands migrated at the same position, which reflects the interaction between these molecules.

We immunoprecipitated insulin from plasma samples using a specific anti-insulin antibody, and then tested for the presence of the alpha subunit portion of the IR (SIR) by Western blot. We observed positive bands ranging in size between 88 kDa and more than 135 kDa (Figure 1B), suggesting the presence of the extracellular domain of the IR as well as some degradation products. We also observed another band of >200 kDa in size that could correspond to a precursor of the IR (12). In native gels, we verified that the bands were detected in the same migrating position for insulin and SIR (Figure 2).

**Figure 3:**
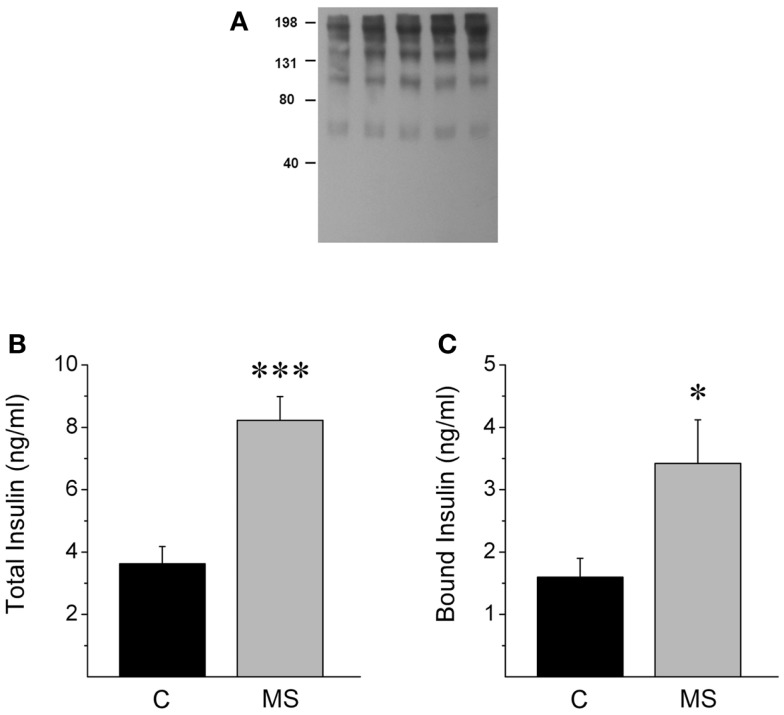
**MS rats show increased insulin binding to plasmatic proteins**. **(A)** Western blot from plasma of control rats (lane 1, 2) and MS rats (3, 4, 5) that were previously separated using different Amicon centrifugal filters and immunoprecipitated with an insulin receptor (alpha-IR) in a native gel, stained with an anti-insulin antibody. Observe that insulin is present in different molecular weight bands, Section “Materials and Methods.” **(B)** Rats with metabolic syndrome (MS) show increased insulin levels when compared to control rats (C). **(C)** MS rats display higher insulin levels in the plasmatic fraction containing proteins above 30 kDa, suggesting higher insulin binding to plasmatic proteins. Bars represent mean ± SEM of control (*n* = 12) and MS rats (*n* = 7), *p < 0.05 and ***p < 0.0001.

We measured insulin levels in control rats and animals that were treated with a hypercaloric diet for 2 months (19). Rats that were fed with 20% sucrose in drinking water (see Materials and Methods). After 2 months, the animals that exhibited insulin levels higher than that of control rats (95% Confidence Interval: 2.41–4.84 ng/ml) were included in the MS group for comparison (Figures 3A,B). For protein-bound insulin determination in rat plasmas, the samples were fractioned using centrifuge filters with a cutoff value of 30 kDa. In both groups, 40% of total insulin was detected in the fraction with proteins above 30 kDa, which indicates that insulin is bound to plasmatic proteins. Furthermore, MS rats displayed a twofold increase in the amount of insulin in the fraction containing proteins above 30 kDa (Figure 3C), which indicates that insulin binding to high molecular weight proteins in plasma is exacerbated in MS.

### The liver as a source of circulating SIR

We hypothesized that hepatocytes were strong candidates to be the tissue that contributes the most to the increased circulating SIR. After being secreted by pancreatic beta-cells, insulin-enriched blood from the pancreas enters the liver through portal circulation and a marked percentage of insulin is retained by this organ. Because hyperinsulinemia increases the cleavage of IRs in hepatocytes (16), we measured the amount of alpha-IR in the conditioned media of primary cultures of rat hepatocytes incubated with different insulin concentrations.

**Figure 4:**
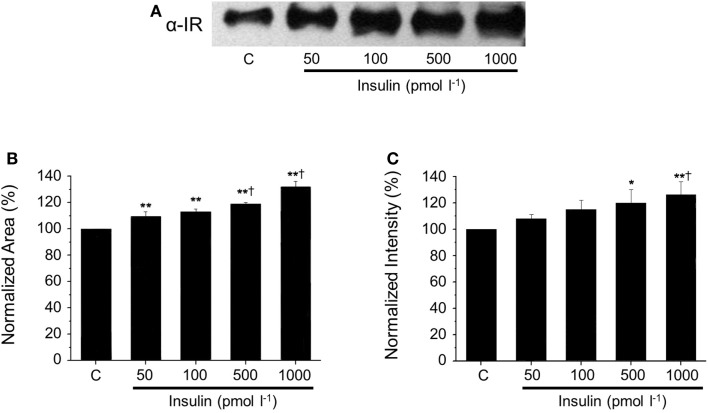
**Dose-dependent SIR release in the supernatant of cultured hepatocytes**. **(A)** Representative western blots against SIR in media collected from cultured hepatocytes incubated with increasing concentrations of insulin. **(B)** Comparison of normalized SIR levels determined by densitometry as the area or **(C)** the intensity of the bands obtained by Western blot. Bars represent mean ± SEM of control and insulin-treated hepatocytes at 50, 100, 500, and 1000 pmol l^−1^ (*n* = 3 each),*p < 0.05 and **p < 0.01 for comparison with respect to control and ^†^*p* < 0.05 with respect to the 50 pmol l^−1^ condition.

After native gel electrophoresis (Figure 4), Western blots from supernatants showed a similar band to the one observed in plasma, which increased proportionally with the concentration of insulin. The increment of SIR levels was insulin dose-dependent (50 pmol l^−1^ to 1 nmol l^−1^), and these concentrations represent a broad of physiological insulin levels. The presence of SIR detected by Western blot was further corroborated using a three-step purification procedure based on the physicochemical properties of SIR.

**Figure 5:**
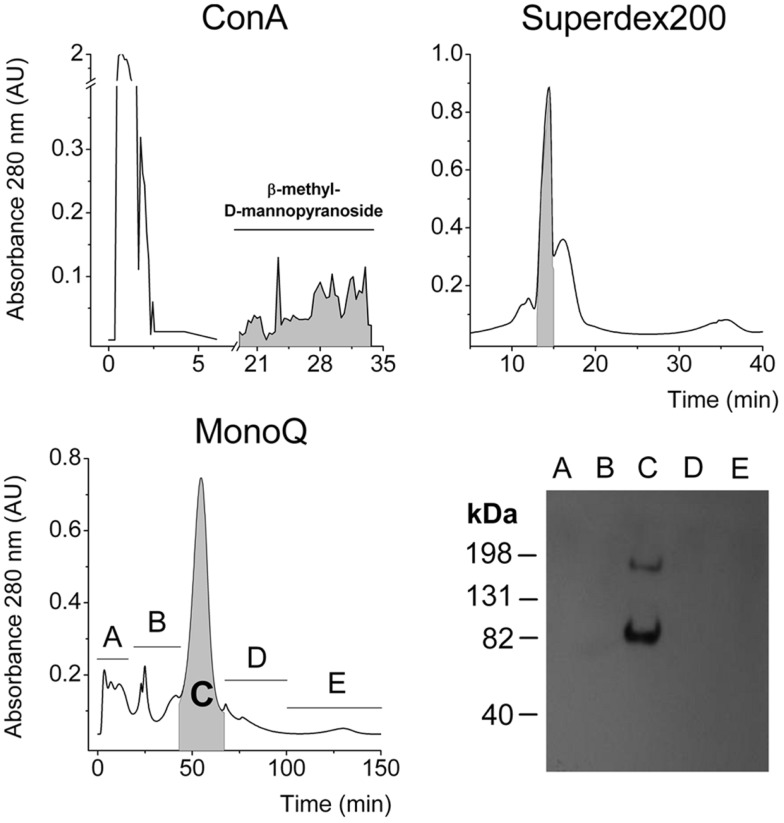
**Three-step purification of SIR from the supernatant of cultured hepatocytes**. Chromatograms from Con-A affinity, gel filtration Superdex 200, and anionic exchange chromatographies. In each purification step, the faded fractions were pooled and assessed for α-IR by Western blot to follow purification. Finally, a representative Western blot of each collected fraction from the last purification step.

The IR is a heavily glycosylated protein (25–28) and binds to Concanavalin-A (Con A) chromatographic columns (1). Gel filtration using a Superdex G-200 column was performed as previously reported (2) and high molecular weight fractions were pooled. Then aliquots were applied to an anion exchange chromatography column, according to the acidic isoelectric point of alpha-IR, which is approximately 4.0 (1, 2). Finally, we detected IR in the major peak, on the basis of the expected molecular weight for the SIR monomer. Another band was observed migrating near 82 kDa and may correspond to an alpha receptor fragment (Figure 5).

### SIR release is mediated by proteases in primary cultures of rat hepatocytes

It has been previously reported for several cell lines and primary cultures of different cell lineages that SIR release is mediated by proteases (17). In order to determine if hyperinsulinemia-induced release is mediated by a similar mechanism, we performed an RHPA in the presence of the plasma protease inhibitor alpha2-macroglobulin.

**Figure 6:**
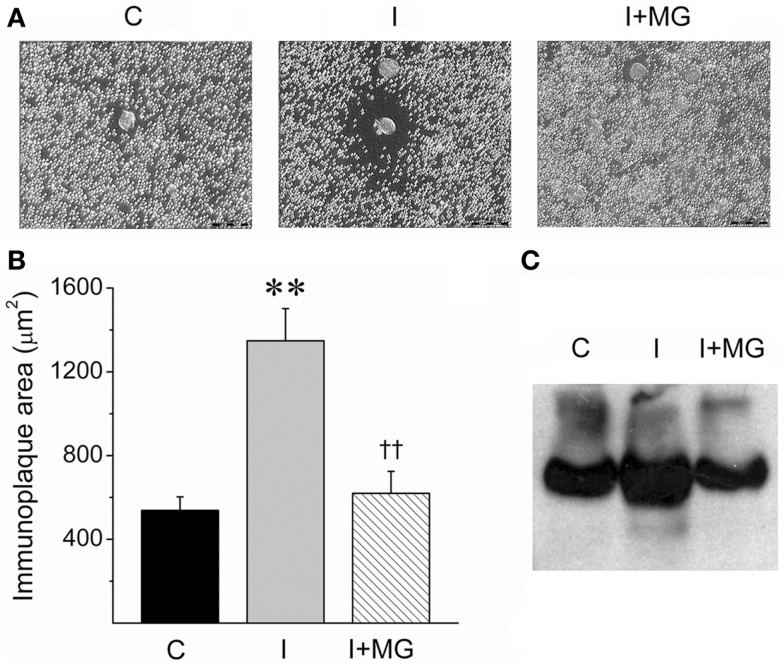
**Insulin-induced SIR cleavage by cultured hepatocytes is reduced by the protease inhibitor alpha2-macroglobulin (MG)**. **(A)** Microphotographs of representative RHPA assays from control (C), 1 nM insulin-treated hepatocytes, (I), and 1 nmol l^−1^ insulin-stimulated plus 1.5 mg/ml alpha2-macroglobulin (I + MG). Scale bars: 50 μm. **(B)** Immunoplaque areas (IPA) as a measure of SIR releasing activity. IPA was calculated after 24 h of incubation in three experiments in duplicate except for MG (*n* = 2 in duplicate). Bars represent mean ± SEM, **p < 0.01 for comparison against control and ^††^*p* < 0.01 for comparison against 1 nmol l^−1^ insulin condition. **(C)** Western blot for SIR of supernatants of hepatocytes cultured in the same conditions of RHPA for 5 days.

We used a novel variant of a RHPA, which was developed to unambiguously identify cells that secrete specific proteins (23). The classic RHPA has been used to detect hormone secretion from single cells after complement-mediated hemolysis in the presence of antigen-antibody complexes (see Materials and Methods). In this modified version, hepatocytes were incubated with protein-A coated erythrocytes that were pre-incubated with a polyclonal antibody against the alpha portion of the IR (see Materials and Methods). Complement was then added, and the hepatocytes that liberated SIR from their membrane were identified with a hemolytic halo (Figure 6A).

The amount of SIR liberated is directly proportional to the area of the immunoplaque surrounding the hepatocytes. The size of the immunoplaques were significantly increased after incubating the hepatocytes with 1 nmol l^−1^ insulin, compared to controls without insulin (Figures 6A,B).

Interestingly, we then included the protease inhibitor alpha2-macroglobulin in the incubation media with 1 nmol l^−1^ insulin and observed that the effect of the insulin was counteracted. This observation is consistent with a protease-mediated activity of SIR release. Similar results were observed when proteins from hepatocyte supernatants were resolved on a native gel and analyzed by Western blot. In this case, the area of the band corresponding to SIR was increased after incubating the hepatocytes with insulin (1 nmol l^−1^) and reduced to basal levels when alpha2-macroglobulin was co-incubated with insulin (Figure 6C). This experiment demonstrates that hepatocytes are a source of SIR, probably by preoteolytic cleavage.

### Plasma SIR levels in rats and human subjects with hyperinsulinemia

**Figure 7:**
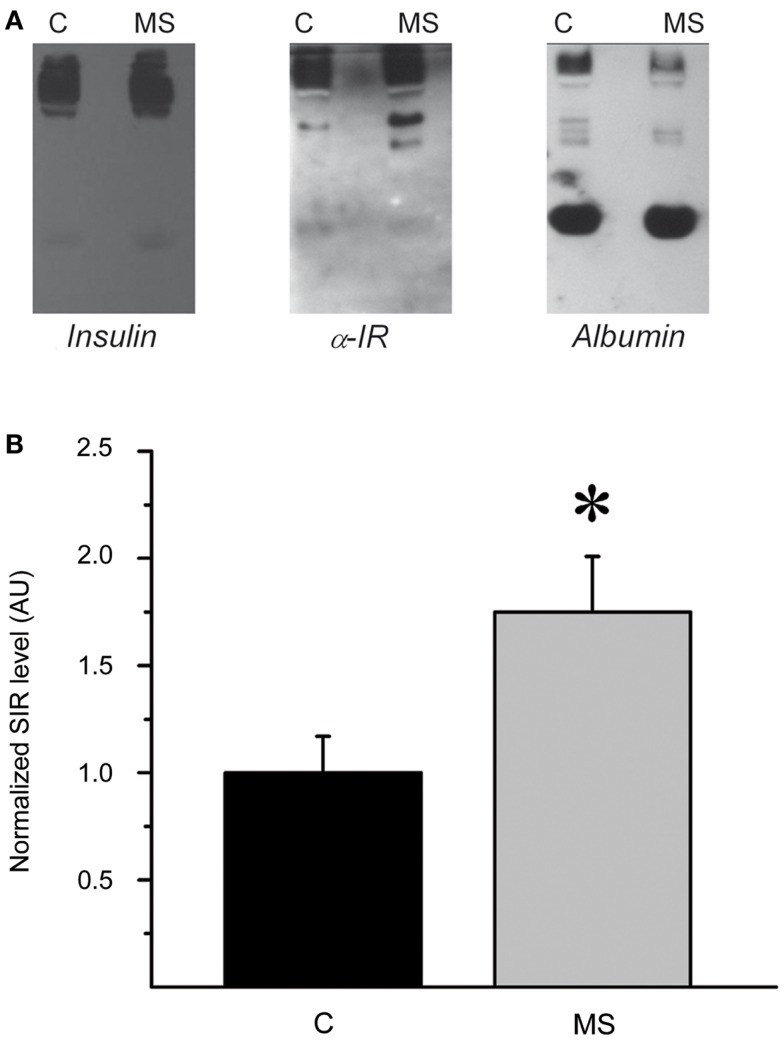
**Plasma SIR is increased in MS rats**. **(A)** Western blots of native gel electrophoresis from representative plasma samples from control (C) and metabolic syndrome (MS) rats that show co-migration of insulin, SIR, and albumin in rat plasma as indicated by the colocalization of all molecules. The intensity and size of insulin and SIR bands are higher in MS rats than controls. **(B)** Comparison of normalized plasma SIR levels determined by densitometry of Western blot bands. Bars represent mean ± SEM of control (C) and MS rats, *p < 0.05 and **p < 0.01.

**Figure 8:**
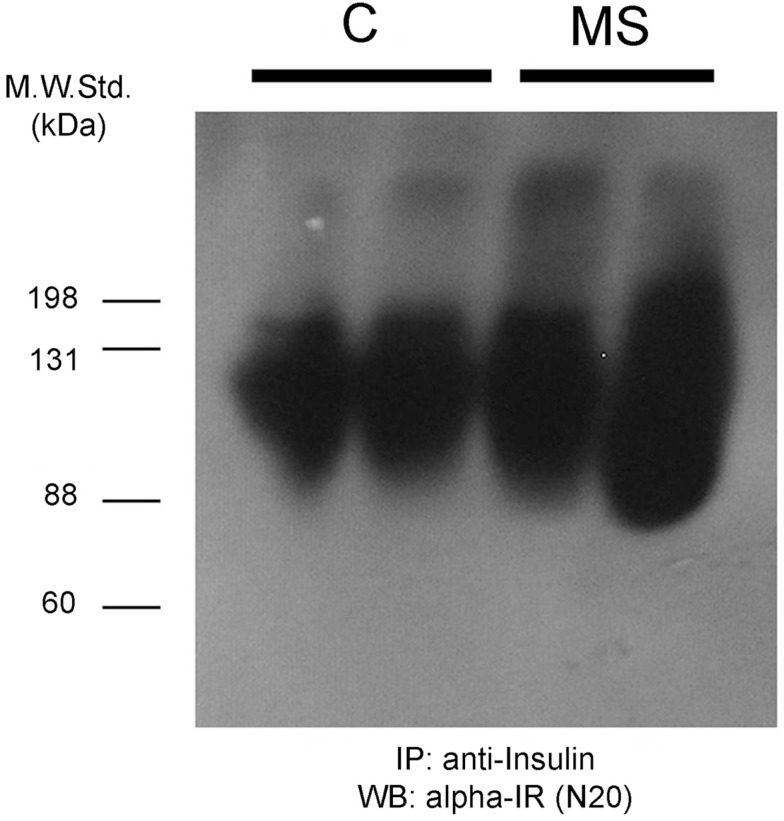
**Insulin-receptor (alpha-IR) staining by western blot after denaturing gel electrophoresis of previously insulin-immunoprecipitated plasma samples from two control rats (C) and two animals with metabolic syndrome (MS)**. As observed, the insulin-receptor positive bands are broader and more intense in the MS rats.

To further characterize the systemic increase of SIR levels in rats with hyperinsulinemia, plasma samples of control and MS rats were analyzed by native electrophoresis gels and Western blots to detect SIR, and albumin. SIR-positive bands in samples from control and MS rats were further quantified and analyzed by densitometry (Figures 7A,B). The level of SIR in the plasma of MS rats was twofold higher than in controls, which may be due to the insulin hypersecretion (Table 2) developed by these animals (Figure 8).

**Table 2:** **Plasma insulin and glucose measurements of control and MS rats**.

Rats	Insulin (μg/l)	Glucose (mg/dl)
Control (*n* = 5)	3.2 ± 0.3	126 ± 24
MS (*n* = 9)	13.4 ± 1.2**	186 ± 21**

**Figure 9:**
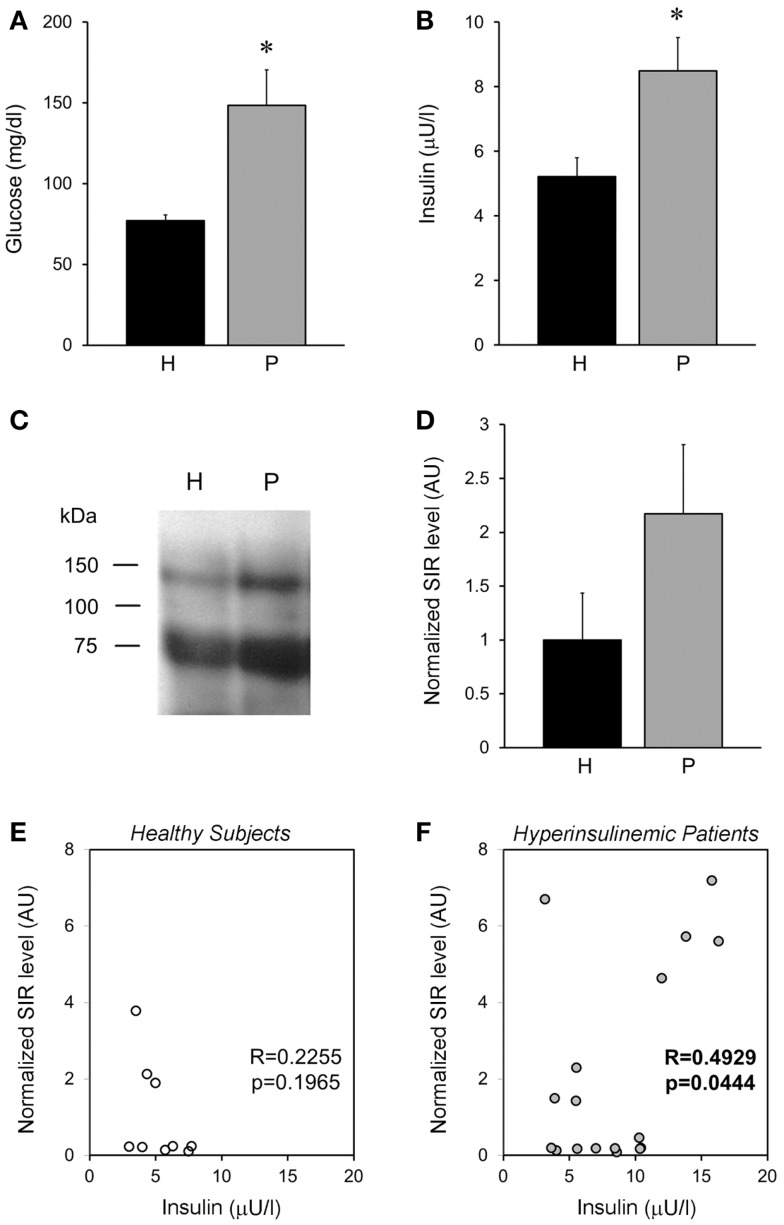
**Plasma SIR is detected in healthy and diabetic patients**. **(A)** Fasting plasma glucose levels and **(B)** insulin, from healthy subjects (H) and patients (P) that present hyperglycemia and hyperinsulinemia. **(C)** Representative Western blots of alpha-IR immunoprecipitated plasma from healthy subjects and hyperglycemic–hyperinsulinemic patients. Note that alpha-IR immunoblotting appears in two bands (close to the expected size for α-IR monomer: 135 kDa, and a fragment around 75 kDa) of a SDS-PAGE gel. **(D)** A tendency of higher SIR levels is observed in the plasma of hyperglycemic–hyperinsulinemic patients. SIR levels were determined by densitometry of the experiments depicted in **(D)** and normalized with respect to the average level of healthy subjects. **(E,F)** Show the correlation between the normalized SIR levels and the plasmatic insulin, which resulted significant for patients. Bars represent mean ± SEM of healthy subjects (*N* = 9) and hyperglycemic–hyperinsulinemic patients (*N* = 17), *p < 0.05.

To characterize if our results obtained in rats were similarly observed in humans. We analyzed plasma obtained from healthy subjects and compared them with hyperglycemic–hyperinsulinemic patients (Figures 9A,B). Interestingly, we observed that healthy subjects showed SIR bands, similar to that observed in the animal model (Figure 9C), moreover in hyperinsulinemic subjects there was a clear tendency to an increase in SIR levels (Figure 9D), although no statistical difference was reached, perhaps because of the high variability of human parameters and the relatively small sample here used. However, we found that insulin levels, although they were not associated with SIR levels in healthy subjects (Figure 9E), they showed a positive correlation in the group of patients (Figure 9F).

## Discussion

In this study we tested the hypothesis that a certain amount of plasma insulin does not travel freely in circulation, but rather it is bound to proteins, predominantly produced by the liver. Insulin binding to proteins would prevent a possible hypoglycemic event and also protects insulin from fast degradation by IDEs. In states of insulin resistance, high insulin levels are detected; however, it is likely that only a small amount of that insulin is free to interact with the respective targets. The insulin-binding proteins that we considered in this study were albumin and a SIR. The observed SIR perfectly coincides with one previously described by the Insulin-Receptor Group in diabetic patients (12). Nevertheless, this study concluded that SIR could be a biological response to hyperglycemia and we demonstrated that it is a response to high insulin levels.

A recent study demonstrated that SIR levels are also correlated with the inflammatory protease circulating granzyme B levels that is increased in type 2 diabetes mellitus (T2DM), and further augmented by obesity in patients (29). Furthermore, these authors found that SIR levels were positively correlated with fasting plasmatic insulin, suggesting a possible association with a hyperinsulinemic state. Here, we proposed and demonstrated that high insulin concentrations promote the liberation of the alpha portion of the IR from hepatocytes, and that a protease inhibitor can prevent this process. The results of *in vitro* experiments were in agreement with the once obtained from whole animal samples, as well as from plasma samples obtained from healthy and hyperinsulinemic patients.

Hyperinsulinemia and insulin resistance are characteristic signs of MS, and we previously developed an animal model that recapitulates the disease course seen in humans. We demonstrate that SIR is elevated in this model and is also associated with hyperinsulinemia, compared to controls.

The presence of a plasma SIR is a phenomenon that has been previously associated with insulin resistance in T2DM in humans and animal models (12, 18, 30). It has been demonstrated that IR cleavage is increased in leukocytes from SHR (17), which is an endogamous strain predisposed to hypertension and insulin resistance, which are two signs of MS. Most of the previous studies focused on the reduced ability of target organs to respond to the circulating insulin levels due to a pathological protease-mediated excision of the cell surface IRs. However, it is important to understand its physiological role.

Spontaneous hypertensive rats show enhanced proteolytic activity in plasma and venules from the mesentery microcirculation compared to normotensive control rats, which is reduced by protease inhibitors. This activity leads to increased cleavage of the IR in SHR leukocytes, which can be attenuated by reducing the matrix metalloproteinase activity with chronic exposure to doxycycline (17). They also demonstrated that the cleavage of the IR leads to a 60% reduction of transmembrane transport of fluorescent glucose (17). Moreover, similar results were obtained in sand rats (*Psammomys obesus*) fed with a hypercaloric diet, for 1 month.

Using an insulin solution that was 1000-fold more concentrated than the one we used, Sanchez-Casas also detected the release of a 120 kDa truncated form of IR from hepatocyte plasma membranes (16). Remarkably, the same study showed a basal degradation of IRs, which is in agreement with our observation of low SIR levels, albeit detectable, in the RHPA and Western blot experiments using hepatocyte supernatants without insulin. However, these authors were uncertain of the *in vivo* relevance of this observation, because they could not detect a significant increment of SIR levels in plasma. This apparent contradiction present in most of the studies reporting elevated SIR levels in T2DM could arise from reliance on the acute hyperinsulinemia model used by Sanchez-Casas et al., which is different from the long-term induction of insulin resistance in diabetic or MS models, as well as the natural, multifactorial development of these pathologies.

Proteinase inhibitors have the ability to decrease insulin-dependent receptor release and proteolysis from plasma membranes in IM-9 cells (13) and hepatocytes (16), respectively. We obtained similar results with one of the tested inhibitors, alpha2-macroglobulin (16), which reduced the SIR immunoplaque areas to basal levels in the insulin-stimulated RHPA, confirming a role for proteases in the process of insulin-induced SIR release from hepatocyte membranes. Hyperinsulinemia also modulates the activities of hepatic matrix metalloproteinases and their tissue inhibitors, which could be related to hepatic fibrosis (31).

It has been shown that intraperitoneal injections of SIR alpha subunits in mice cause a significant increase in blood-glucose levels as well as glucose intolerance (18). We observed that in an MS model in rats, SIR levels were also increased. In a previous study, our group demonstrated that this animal model develops hyperinsulinemia and insulin resistance after 2 months under a hypercaloric diet (19). Interestingly, we also observed higher levels of SIR in hyperinsulinemic patients, compared to healthy subjects. Together, these data suggest that SIR levels may be a marker of insulin resistance.

**Figure 10:**
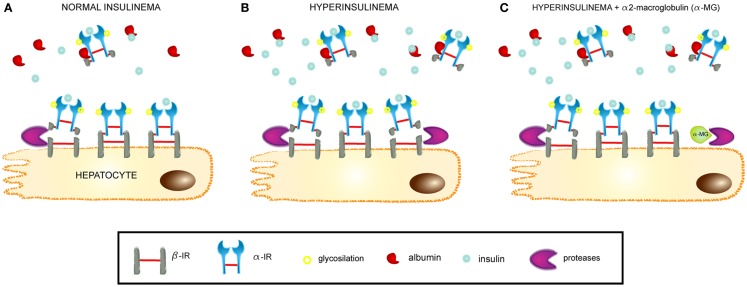
**Mechanism of SIR release from hepatocytes**. **(A)** At normal insulin levels, SIR is cleaved from hepatocyte membranes by proteases and passes into the bloodstream, where it retains the ability to bind insulin and also albumin. SIR acts as a physiological carrier for insulin. **(B)** At hyperinsulinemic conditions, SIR is released and the levels increase in plasma, where it can contribute to insulin resistance by its high affinity binding of the hormone. **(C)** Proteinase inhibitors, such as alpha2-macroglobulin, modulate SIR release by impeding the cleavage of the insulin receptor within a segment of the beta-domain.

A general overview of the novel pathophysiological mechanism leading to insulin resistance, as proposed in the present work, is depicted in Figure 10. Briefly, hyperinsulinemia increases the proteolytic activity on surface IRs in hepatocyte membranes. This leads to the release of these receptors into the bloodstream and contributing to insulin resistance through a dual mechanism, first by decreasing the density of IRs present on cells and the insulin responsiveness, and second by sequestering the circulating insulin through SIR, which competes with the membrane-bound receptors for the available insulin in circulation.

These results open a new field in the study of MS, as we show that the plasma SIR is an important regulator of free insulin in blood under both physiological and pathological conditions. Moreover, we show that hyperinsulinemia is a reflection of high amounts of insulin bound to proteins at a higher proportion than free insulin.

## Conflict of Interest Statement

The authors declare that the research was conducted in the absence of any commercial or financial relationships that could be construed as a potential conflict of interest.
